# Signal Enhancement in the HPLC-ESI-MS/MS analysis of spironolactone and its metabolites using HFIP and NH_4_F as eluent additives

**DOI:** 10.1007/s00216-017-0255-4

**Published:** 2017-02-21

**Authors:** Kalev Takkis, Rudolf Aro, Lenne-Triin Kõrgvee, Heili Varendi, Jana Lass, Koit Herodes, Karin Kipper

**Affiliations:** 1 Institute of Chemistry, University of Tartu, 14a Ravila Street, Tartu, 50411 Estonia; 2 Children’s Clinic, Tartu University Hospital, N. Lunini 6, Tartu, 51014 Estonia; 3 Institute of Biomedicine and Translational Medicine, Department of Pharmacology, University of Tartu, Ravila 19, Tartu, 50411 Estonia; 4 Pharmacy Department, Tartu University Hospital, L. Puusepa 8, Tartu, 51014 Estonia; 5 Institute of Biomedicine and Translational Medicine, Department of Microbiology, University of Tartu, Ravila 19, Tartu, 50411 Estonia; 6 Paediatric Infectious Diseases Research Group, Institute for Infection and Immunity, St. George’s, University of London, Cranmer Terrace, London, SW17 0RE UK

**Keywords:** Spironolactone, Ammonium fluoride, Signal enhancement, Liquid chromatography-mass spectrometry, Ionization efficiency, Hexafluoroisopropanol

## Abstract

This paper describes an LC-MS/MS method to determine the concentration of spironolactone and its metabolites 7-alpha-methylthiospironolactone and canrenone in blood plasma samples. The resulting assay is simple (using protein precipitation for sample preparation) and sensitive (the lower limit of quantification is close to 0.5 ng/ml) while requiring only 50 μl of plasma, making it especially suitable for analyzing samples obtained from pediatric and neonatal patients where sample sizes are limited. The sensitivity is achieved by using ammonium fluoride as an eluent additive, which in our case amplifies the signal from our analytes in the plasma solution on average about 70 times. The method is fully validated according to the European Medicines Agency’s guideline and used for the measurement of pediatric patients’ samples in clinical trials for evaluating oral spironolactone’s and its metabolites’ pharmacokinetics in children up to 2 years of age.

## Introduction

**Fig. 1:**
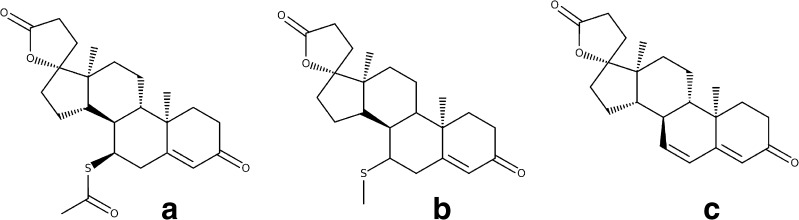
Chemical structures of spironolactone (**a**), 7-alpha-methylthiospironolactone (**b**), and canrenone (**c**)

Spironolactone (Fig. 1) is a synthetic steroidal antimineralocorticoid agent with a structure resembling that of the natural adrenocorticoid hormone, aldosterone. Spironolactone competes with aldosterone on aldosterone-sensitive Na^+^/K^+^ channels in the distal tubule of the nephron, thereby increasing the secretion of water and sodium while decreasing the excretion of potassium [1]. It is rapidly and extensively metabolized in the liver to at least 17 metabolites, with canrenone, 7-alpha-methylthiospironolactone (two pharmacologically active spironolactone metabolites, Fig. 1), and 6-beta-hydroxy-7-alpha-methylthiospironolactone as the main ones [2]. Spironolactone has been widely used for decades and its side effects have been studied and documented [3–8], but despite being used also in children, the dosing recommendations are based on clinical trials performed in the adult population and/or expert opinion and clinical experience. As such, studies evaluating the pharmacokinetic profile of spironolactone in pediatric age groups contribute to the refinement of evidence-based dosing recommendations in children.

Analytical methods measuring spironolactone and its metabolites in serum or plasma published in previous studies have commonly employed separation by reversed phase liquid chromatography (RPLC), followed by detection either by UV/VIS spectrophotometry or mass spectrometry. The latter is a more popular choice, for obvious reasons: lower detection limits can be achieved and the interference from the complex sample matrix is decreased. This is especially true for tandem mass spectrometric detection where the fragmentation of molecules creates a characteristic fingerprint and thereby greatly increases selectivity. Employing these methods has resulted in quantitation limits for human plasma samples as low as 2 ng/ml [9]. However, these methods often require either complex or time-consuming sample preparation steps such as solid phase extraction, or rather large sample sizes, ranging from 100 to 1000 μl [9–13]. Since the age and condition of the target population implied much smaller available plasma volumes, an effort was made to increase the sensitivity of the assay while keeping it as simple as possible.

One particularly interesting way to increase the assay’s sensitivity is through eluent additives which due to various known or unknown mechanisms improve an analyte’s ionization and increase the analytical signal. In our case—steroid-like molecules—an additive that has emerged from the literature which significantly enhances their ionization is ammonium fluoride [14–16]. The suitability of NH_4_F as an eluent additive in LC-MS methods has been thoroughly evaluated [14–20] and it has been found that the signal-enhancing effect is dependent on the analyte’s chemical structure, with signal enhancement and signal suppression both being possible [20]. The mechanism behind the sensitivity improvement in the negative ionization mode probably stems from the fluoride ions’ strong basicity in the gas phase, allowing it to capture protons from the neutral analytes, resulting in HF [14] and the forming of both [M+F]^−^ ions and [M+FHF]^−^ clusters. Signal enhancement due to ammonium fluoride in the positive ESI mode has been demonstrated for some organic acids by aqueous normal phase chromatography but the mechanism remains largely unknown [19].

The aim of the present study was to develop and validate the selective and sensitive HPLC-ESI-MS/MS method for the determination of spironolactone, 7-alpha-methylthiospironolactone, and canrenone from small volume blood plasma samples and to evaluate the suitability of NH_4_F as an eluent additive for the analytes.

## Materials and methods

The clinical study protocol was approved by the Research Ethics Committee of the University of Tartu (249/M-12, 15.05.2015). For the method development and validation, no clinical study samples were analyzed. Blood plasma with EDTA and Citrate Phosphate Dextrose Solution (CPD) for method development and validation was purchased from the Blood Centre of Tartu University Hospital.

### Chemicals

Spironolactone and canrenone were purchased from Sigma Aldrich (St. Louis, MO, USA), 7-alpha-methylthiospironolactone from PharmaSynth (Tartu, Estonia), and spironolactone-D_3_ (used as an internal standard, IS) from TLC Pharmaceutical Standards (Aurora, Ontario, Canada). LC-MS-grade methanol, tetrahydrofuran (THF), NH_4_F, and 1,1,1,3,3,3-hexafluoro-2-propanol (HFIP) were obtained from Sigma Aldrich (St. Louis, MO, USA). Water was purified (18.2 MΩ·cm at 25 °C and a TOC value in the range 2–5 ppb) in house using a Millipore Advantage A10 system from Millipore (Bedford, USA).

### Sample preparation

Plasma samples, calibrators, and quality control samples were stored at −70 °C. For analysis, the samples were thawed at room temperature. Once at room temperature, the samples were mixed on an Eppendorf MixMate and 50 μl of plasma was transferred into a 2-ml polypropylene Eppendorf tube. One hundred thirty microliters of methanol and 50 μl of IS (spironolactone-D_3_) solution in methanol were added. The samples were then shaken for 2 min at 2050 rpm and subsequently centrifuged for 5 min at 27,500×*g* and 4 °C.

### Chromatographic conditions

The Agilent 1290 Infinity (Santa Clara, USA) HPLC system with a binary pump, a thermostated column compartment, and an autosampler (thermostated at 4 °C) was used. For the chromatographic separation, the YMC-Triart C18 analytical column (150 × 3.0 mm, 3 μm) was used. To protect the column, an in-line filter and the YMC-Triart C18 guard column (10 × 3.0 mm, 3 μm) were installed in front of the analytical column. During analysis, the column was thermostated at 40 °C.

The aqueous phase of the eluent comprised 5 mM HFIP and 2.5 mM NH_4_F in a mixture of THF and water (36:1000). For analysis, isocratic elution with methanol (35:65) was used. At the end of every run, the column was flushed with pure methanol.

### Mass spectrometry

The Agilent 6495 Triple Quadrupole (Santa Clara, USA) served as a mass analyzer. The heated ESI ionization source (Agilent JetStream Technology) in the positive mode was used. Nitrogen served as a nebulizing, sheath, drying, and collision gas. Sheath gas temperature was set to 350 °C and drying gas temperature to 250 °C. The data were collected in the MRM mode; the transitions monitored and the respective collision energies are listed in Table 1. The LC-MS system was controlled with the Agilent MassHunter Workstation software version B.07.00. For peak integration and quantitative calculations, the Agilent MassHunter Quantitative Analysis software version B.07.00 was used.

**Table 1:** MRM transitions used for analysis with collision energies (CE)

Compound	Precursor ion	Product ion			
		Quantifier		Qualifier	
	*m/z*	*m/z*	CE, V	*m/z*	CE, V
Spironolactone	341	107	38	341	2
Canrenone	341	107	38	341	2
7-Alpha-methylthiospironolactone	389	341	14	323	14
Spironolactone-D_3_	344	107	32	341	2

### Calibration

Matrix-matched calibration was used. Stock solutions at about 1 mg/ml were prepared in methanol and stored at −70 °C. For spiking, stock solutions were diluted in water. A total of eight calibration points were prepared in the blood plasma with concentrations of approximately 0.5, 1, 2, 6, 20, 50, 150, and 250 ng/ml. Quality control samples were prepared from different stock solutions at four levels (approximately 0.6 ng/ml (lower limit of quantification, LLOQ), 4 ng/ml (low), 80 ng/ml (medium), and 220 ng/ml (high) plasma concentration). Linear regression with 1/*x* ^2^ weights was used for creating calibration curves.

### Method validation

The method was fully validated according to the European Medicines Agency’s (EMA) guideline [21]. Linearity was evaluated by comparing the experimental and measured concentrations of the calibration solutions on different days during the validation. Inter- and intra-day accuracy and precision were determined by running a batch of the calibration curve and quality controls on three different days; selectivity was evaluated by injecting blank plasma samples from six healthy volunteers; carry-over was calculated from blank plasma samples immediately following the highest calibration injection. Matrix effects were evaluated as described by Matuszewski et al. [22] by comparing the post-extraction spiked plasma samples (from six plasma lots obtained from individual donors [21]) and standard solutions in neat solvent at high and low concentration levels. Stability was determined in short-term, freeze, and thaw cycles, on-instrument, and long-term forms. For short-term stability evaluation, the spiked samples were kept for 24 h at 20 ± 2 °C; for on-instrument stability in the autosampler, the prepared samples were kept at 4 °C for 25 h; a total of three freeze and thaw cycles were applied for freeze and thaw stability, and for long-term stability, the spiked plasmas were stored for 5 months at −70 °C.

## Results

### Limit of quantification, linearity, carry-over, accuracy, and precision

The final method is a fairly standard LC-MS/MS method with an aqueous/methanol mobile phase and a well-established protein precipitation-based sample preparation. LLOQ were 0.53 ng/ml for spironolactone, 0.52 ng/ml for 7-alpha-methylthiospironolactone, and 0.55 ng/ml for canrenone, lower than reported in the literature before [9].

The calibration set comprised eight calibration points (prepared in duplicates), blank and double blank (blank plasma without IS). The back-calculated concentrations from the curve remained within ±15% of accuracy. Linearity, expressed as the coefficient of determination (*R* ^2^), was >0.93 for all analytes. Carry-over did not present a problem as all analytes were undetectable from blank injections after the highest calibrator, and with regard to selectivity, no interfering peaks for analytes and the internal standard were found in the double blank samples. The data for accuracy and precision are presented in Tables 2 and 3, respectively.

**Table 2:** Accuracy data (*n* = 5 for within-run accuracy; *n* = 3 for between-run accuracy) in spiked plasma samples

Analyte	Accuracy (%)			
	At the LLOQ level		Low, medium, and high concentration QC-s	
	Within-run	Between-run	Within-run	Between-run
Spironolactone	−19.7…13.5	−19.7…13.5	−7.4…6.4	−7.4…5.5
7-Alpha-methylthiospironolactone	−5.9…8.8	−5.9…1.5	1.1…11.4	2.1…11.4
Canrenone	−10.9…18.2	−5.1…18.2	0.6…14.7	0.6…11.5

Quality control samples at low (4 ng/ml), medium (80 ng/ml), and high (220 ng/ml) concentration

**Table 3:** Precision data (*n* = 5 for within-run precision; *n* = 3 for between-run precision) in spiked plasma samples

Analyte	Precision (CV%)			
	At the LLOQ level		Low, medium, and high concentration QC-s	
	Within-run	Between-run	Within-run	Between-run
Spironolactone	8.9	7.9	3.2	2.7
7-Alpha-methylthiospironolactone	4.0	2.1	2.7	2.1
Canrenone	7.8	7.5	2.7	2.1

Quality control samples at low (4 ng/ml), medium (80 ng/ml), and high (220 ng/ml) concentration

### Stability

Stability data for freeze and thaw cycles, short-term, on-instrument, and long-term storage are presented in Table 4. To summarize the results, while the analytes are not strongly affected by freeze and thaw cycles and both long-term and on-instrument stability are satisfactory, the plasma stability at room temperature is rather poor. The corresponding increase in canrenone content suggests that some of the spironolactone and 7-alpha-methylthiospironolactone might transform into canrenone. Degradation of spironolactone in rat plasma has been noted before by Tokumura et al. [23], and the dependence on temperature and pH as well. This emphasizes the necessity of careful planning of sample collection in the hospital setting. The decrease of spironolactone content at room temperature indicates the need for controlled sampling conditions and quick sample processing methods to minimize the time samples spend in ambient conditions.

**Table 4:** Stability results. Evaluated for QC samples (*n* = 5)

Stability (%) (SD)	Spironolactone		7-Alpha-methylthiospironolactone		Canrenone	
	Low concentration (0.96 ng/ml)	High concentration (69.04 ng/ml)	Low concentration (0.95 ng/ml)	High concentration (67.80 ng/ml)	Low concentration (0.99 ng/ml)	High concentration (71.12 ng/ml)
Freeze-thaw (3 cycles)	92.5 (5.1)	86.0 (2.6)	94.0 (4.6)	92.6 (2.2)	91.8 (10.6)	98.9 (4.4)
Short term (24 h, 20 °C)	52.4 (6.3)	39.1 (2.2)	83.6 (3.3)	81.4 (4.2)	113.4 (5.0)	130.3 (7.6)
Long term (∼5 months, −70 °C)^a^	110.2 (2.1)	107.4 (0.8)	107.9 (2.9)	110.9 (1.5)	114.0 (5.8)	108.9 (0.6)
On-instrument (25 h, 4 °C)^b^	97.8 (1.9)		96.8 (2.7)		99.2 (3.4)	

^a^Low and high have different values here: 0.60 and 224.70 ng/ml for spironolactone, 0.57 and 211.71 ng/ml for 7-alpha-methylthiospironolactone, and 0.59 and 220.53 ng/ml for canrenone

^b^Calculated over 16 calibration points (0.5…250)

### Matrix effects

Matrix effects were evaluated for high and low analyte concentrations calculating the matrix factors (MF) in six lots of blank plasmas from individual donors. The results are presented in Table 5. While the IS-normalized MF for spironolactone lie in a very reasonable range of 97.3–102.3%, the signal of the other two analytes appears to be amplified in the plasma. The analysis of peak areas revealed that while the intensities of 7-alpha-methylthiospironolactone and canrenone remain about the same in both pure solutions and post-extraction samples (the matrix effect around 100%), the signals from spironolactone and its deuterated analogue are somewhat suppressed (ranging 57.0–67.4 and 56.9–68.8%, respectively). Since the same internal standard is used for all analytes, the reduction in the IS’s signal is responsible for the apparent increase in 7-alpha-methylthiospironolactone’s and canrenone’s intensities. The matrix-matched calibration in plasma was used to take the matrix effect into account for all analytes.

**Table 5:** Matrix effects for spironolactone, 7-alpha-methylthiospironolactone, canrenone, and IS

Concentration	Matrix	MF (%)				IS normalized MF (%)		
		Spironolactone	7-Alpha-methylthiospironolactone	Canrenone	IS	Spironolactone	7-Alpha-methylthiospironolactone	Canrenone
Low, 3 ng/ml	Matrix 1	58.2%	74.2%	72.8%	56.9%	102.3%	130.4%	128.1%
	Matrix 2	58.3%	75.7%	72.2%	57.9%	100.7%	130.7%	124.8%
	Matrix 3	57.3%	73.9%	70.5%	58.2%	98.3%	126.8%	121.0%
	Matrix 4	57.0%	69.9%	70.2%	57.9%	98.3%	120.7%	121.2%
	Matrix 5	60.2%	67.6%	69.2%	61.0%	98.6%	110.9%	113.5%
	Matrix 6	59.2%	71.6%	68.8%	59.8%	99.0%	119.6%	114.9%
	CV%	2.1%	4.2%	2.3%	2.6%	1.6%	6.2%	4.7%
High, 200 ng/ml	Matrix 1	64.8%	73.1%	79.5%	64.6%	100.4%	113.2%	123.1%
	Matrix 2	66.7%	69.3%	77.6%	65.8%	101.3%	105.3%	117.9%
	Matrix 3	67.4%	67.4%	77.1%	68.8%	98.0%	98.0%	112.1%
	Matrix 4	66.1%	66.3%	72.7%	67.9%	97.3%	97.6%	106.9%
	Matrix 5	66.7%	68.4%	72.9%	67.2%	99.2%	101.8%	108.6%
	Matrix 6	67.1%	68.4%	77.1%	67.3%	99.7%	101.7%	114.5%
	CV%	1.4%	3.4%	3.6%	2.3%	1.5%	5.6%	5.3%

## Discussion

When using concentrated solutions, interference from IS to spironolactone’s transitions and vice versa was significant. Fortunately, because the samples turned out to be sufficiently dilute, the effect was not significant in our case. Only the highest calibration points (∼250 ng/ml) were noticeably affected, but since 1/*x* ^2^ weighted regressions were used, it did not have a detrimental effect on linearity. However, on the basis of our experience, using this particular internal standard at higher concentrations will require some measures to be taken to counter this effect.

**Fig. 2:**
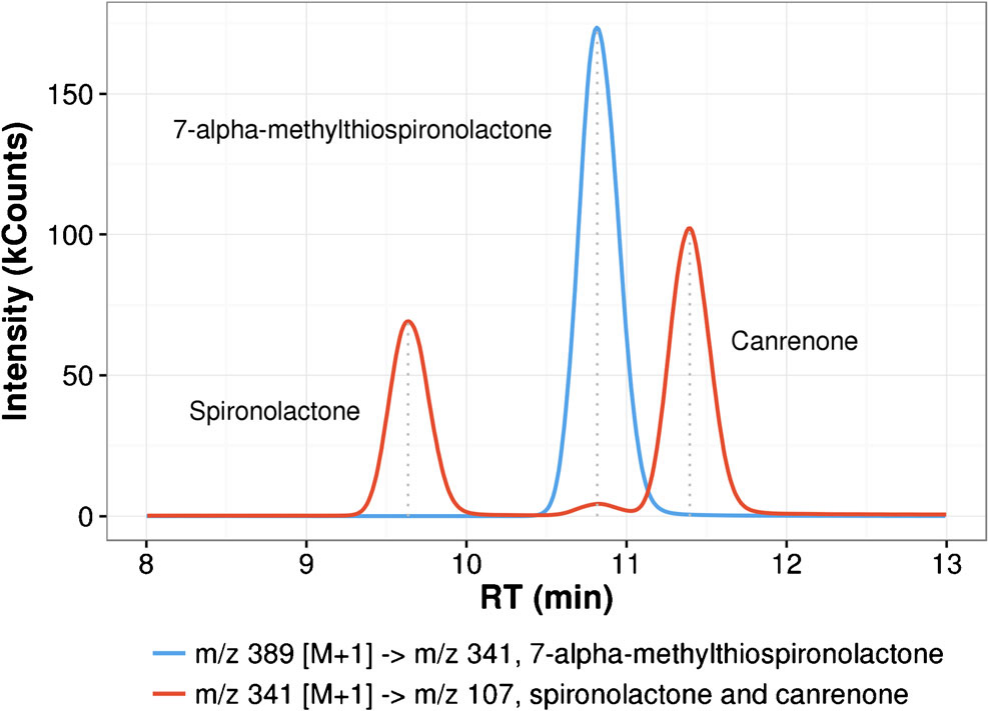
A sample chromatogram of a spiked plasma (about 50 ng/ml) injection. Analytes at their quantitative transitions. Notice the small mound caused by 7-alpha-methylthiospironolactone at spironolactone’s and canrenone’s transition points

Another problem we faced was the question of chromatographic separation. All our analytes have similar p*K*_a_ values and similar overall hydrophobicity, meaning that several conventional RPLC columns failed to provide sufficient separation, the biggest problem here being the overlap between 7-alpha-methylthiospironolactone and canrenone. Various common solvent mixtures used in LC-MS were tested, but sufficient separation was not achieved until THF was added to the mixture. The effect of THF in our case was the acceleration of the retention of spironolactone and 7-alpha-methylthiospironolactone, resulting in a cleaner separation from canrenone (Fig. 2). As to why the separation was deemed so crucial in MS/MS analysis—it appeared that two out of our three analytes largely decomposed (in-source CID) in the ESI source or ion transport and declustering region of MS preceding the first quadrupole. Spironolactone lost its thioacetyl- and 7-alpha-methylthiospironolactone its thiomethyl group, rendering them indistinguishable by *m*/*z* from canrenone with molecular ions being hardly detectable. The best MS/MS transition (Table 1) for spironolactone and canrenone found (as already reported by others [12, 13]) was *m/z* 341 [M+H]^+^ ->*m/z* 107. Although 7-alpha-methylthiospironolactone was quantified on different transition, it was still present in noticeable amounts on this one.

**Fig. 3:**
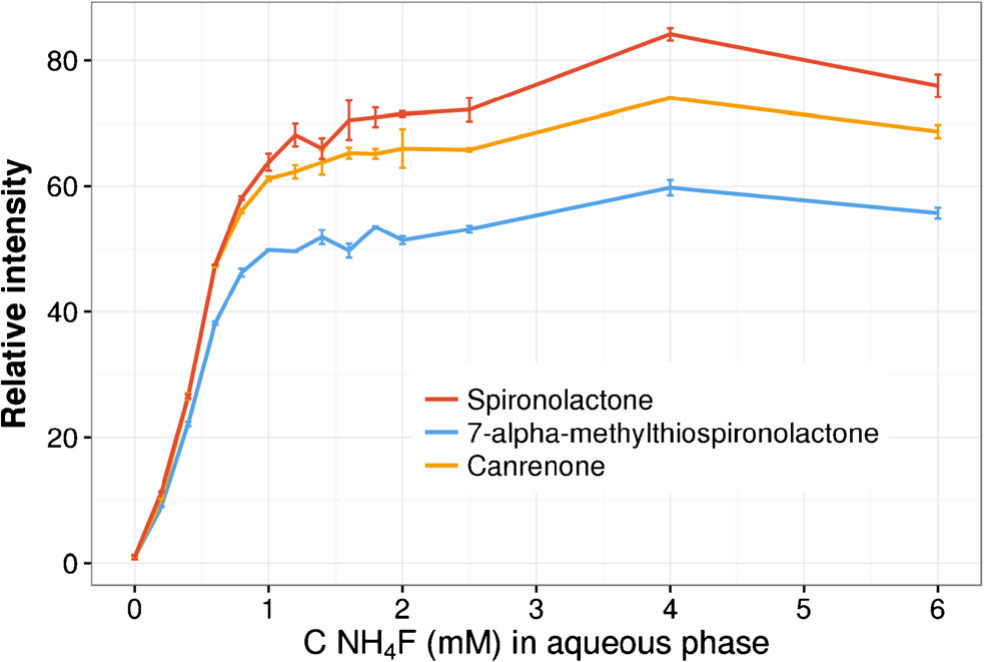
Relative increase in intensity with increasing the NH_4_F concentration in an aqueous phase

The most problematic issue, however, was the ionization of analytes. The difficulties ionizing steroid-like molecules have been noted previously [14–16], and as a potential solution to help them acquire a charge more easily, the eluent additive ammonium fluoride has been proposed [14–16]. NH_4_F has also been found to aid the ionization of various other molecules [14–20], but with some exceptions [19] the increased ionization efficiency has been reported for negative mode. For our system, spironolactone and its two metabolites, we observed a strong increase in ionization efficiency in the positive mode. Our experiments to find an optimal NH_4_F concentration revealed that with plasma sample injections, the signal with ammonium fluoride was on average about 70 times higher than without it [24] (Fig. 3). To the best of our knowledge, NH_4_F has not been used as an eluent additive for spironolactone, 7-alpha-methylthiospironolactone, or canrenone before.

## Conclusions

The development of LC-MS/MS methods to determine poorly ionizable compounds from limited volume samples (pre-clinical studies or clinical trials on pediatric patients) is challenging. The use of eluent additives such as NH_4_F can help boost ionization and enhance the signal; in this case, the increase was about 70 times. The lowest limit of quantification of 0.5 ng/ml was demonstrated using only 50 μl of blood plasma, making the method suitable for use in pediatric patients’ samples.
